# Supplementary material for: Development of the 99mTc-Labelled SST2 Antagonist TECANT-1 for a First-in-Man Multicentre Clinical Study
Source: Pharmaceutics. 2023 Mar 9;15(3):885. doi: 10.3390/pharmaceutics15030885 (PMC10053408; doi:10.3390/pharmaceutics15030885)

pharmaceutics

Supplementary

Supplementary Materials: Development of the ^{99m}Tc-labelled SST$_2$-receptor antagonist TECANT-1 for a first-in-man multi-centre clinical study

Novak Doroteja, Barbara Janota, Anton Amadeus Hörmann, Agnieszka Sawicka, Marko Kroselj, Alicja Hubalewska, Melpomeni Fani, Renata Mikolajczak, Petra Kolenc, Clemens Decristoforo, Piotr Garnuszek

Table of content

Table S1. *Properties of the radio-(RP)HPLC system.* 2
Table S2. *Properties of the iTLC system used for determination of the ^{99m}Tc-colloid species.* 2
Table S3. *Properties of the iTLC system used for determination of the [^{99m}Tc]TcO$_4^-$.* 2
Table S4. *Lyophilisation scheme.* 3
Table S5. *Summary of all tested parameters in development batches 05-07/20.* 3
Table S6. *Summary of initial [^{99m}Tc]Tc-labelling experiments of 2-vial kits (radiolabelling: RT, 30 min, 400 MBq [^{99m}Tc]TcO$_4^-$).* 4
Table S7. *Mean value (n=3) of RCP determined by radio-(RP)HPLC and iTLC in dependence of the TECANT-1 content in development batches 05-07/20 (radiolabelling: RT, 30 min, ~600 MBq [^{99m}Tc]TcO$_4^-$).* 4
Table S8. *Direct comparison of labelling batch 01B/21 using two different ^{99}Mo/^{99m}Tc-generators.* 4
Figure S1: *Representative radio-TLC chromatogram of [^{99m}Tc]Tc-TECANT-1 using 5 M ammonium acetate:methanol (1:1)* …5

Table S1. *Properties of the radio-(RP)HPLC system.*

**radio-(RP)HPLC system**			
Column	Kinetex® 2.6 µm PS C18, 100x2.1 mm 100 Å (Phenomenex, USA)		
Detection	UV-Vis (λ=220 nm): for identity and TECANT-1 assay		
	UV-Vis (λ=220 nm) and radiodetection: radiochemical purity determination		
Injection volume	10 µL		
Sample preparation	1:5 dilution of neutralized radiolabelling solution with water for injection		
Eluents	A: 0.1% TFA in H_2O		
	B: 0.1% TFA in ACN		
Gradient	Time (min)	A (%)	B (%)
	0	85	15
	5	65	35
	6	20	80
	6.1	85	15
	10	85	15
Flow	0.4 mL/min		
Run time	10 min		

Table S2. *Properties of the iTLC system used for determination of the ^{99m}Tc-colloid species.*

**iTLC system**	
Stationary Phase	iTLC – SG (Agilent Technology): approx. 9×1 cm
Mobile Phase	5 M ammonium acetate buffer:methanol (1:1) (*v*/*v*)
Radiodetection	Radio detector: TLC plate reader (ScanRam, LABLogic, Broomhill, United Kingdom)
	Radiochromatography software: Laura (LABLogic, Broomhill, United Kingdom)
Sample volume	5 µL
Sample preparation	no specific sample preparation
Developing distance	80 mm
Analysis time	approx. 10 min

Table S3. *Properties of the iTLC system used for determination of the [^{99m}Tc]TcO_4^-.*

**iTLC system**	
Stationary Phase	iTLC – SG (Agilent Technology): approx. 9×1 cm
Mobile Phase	methyl ethyl ketone (MEK)
Radiodetection	Radio detector: iTLC plate reader (ScanRam, LABLogic, Broomhill, United Kingdom)
	Radiochromatography software: Laura (LABLogic, Broomhill, United Kingdom)
Sample volume	5 µL
Sample preparation	no specific sample preparation
Developing distance	80 mm
Analysis time	approx. 10 min

Table S4. *Lyophilisation scheme.*

Parameter	Section												
	Freezing				Drying						Second drying		
Time [h]	2	1	1	2	0.25	2	0	1	5	5.75	0.3	0.6	2
T [°C]	-40	-40	-35	-35	-35	-30	-20	-20	0	25	25	25	25
Pressure [mbar]	/	/	/	/	0.22	0.22	0.22	0.22	0.22	0.22	0.01	0.01	0.01

Table S5. *Summary of all tested parameters in development batches 05-07/20.*

Batch No.	TECANT-1 [µg]	Number of labellings	Activity added [MBq]	Labelling pH	Incubation time [min]	RCP [%]
05/20	10	1	623	11	10	98.8
		5	623-701	11	20	95.5±0.7
		5	310-701	11	30	93.9±4.8
06/20	15	5	511-712	11	10	97.7±0.1
		5	511-712	11	20	96.4±0.7
		5	511-840	11	30	95.5±1.2
		1	500	8	10-30	76.3-98.0
07/20	50	5	555-640	11	10	93.8±2.0
		5	555-640	11	20	94.7±2.6
		5	555-1200	11	30	95.8±2.2

Table S6. *Summary of initial [^{99m}Tc]Tc-labelling experiments of 2-vial kits (radiolabelling: RT, 30 min, 400 MBq [^{99m}Tc]TcO$_4^-$).*

	Production details						Quality control results		
	Vial 1					Vial 2			
**Batch No.**	**TECANT-2 [µg]**	**$SnCl_2$ x $2H_2O$ [µg]**	**Sodium citrate [mg]**	**Na_2HPO_4 x $12H_2O$ [µg]**	**NaOH [mg]**	**NaH_2PO_4 × 2 H_2O [mg]**	**% [^{99m}Tc]Tc–TECANT-2 (HPLC) [%]**	**% ^{99m}Tc-colloid species [%]**	**RCP [%]**
02a/20	10	15	0.13	1.77	0.4	1.6	70.5±0.0	1.6±0.2	68.9
02b/20	15	15	0.13	1.77	0.4	1.6	83.5±0.1	4.3±0.5	79.2
02c/20	15	15	0.13	1.77	0.4	1.6	86.7±0.2	3.2±0.6	83.5
03/20	15	15	0.13	1.77	0.4	1.6	79.2±0.3	5.2±1.1	74.0
04/20	10	25	0.13	1.77	0.4	1.6	57.7±0.2	n.d.	57.7

Table S7. *Mean value (n=3) of RCP determined by radio-(RP)HPLC and iTLC in dependence of the TECANT-1 content in development batches 05-07/20 (radiolabelling: RT, 30 min, ~600 MBq [^{99m}Tc]TcO$_4^-$).*

	**Production details**	**Quality control results**			
**Batch No.**	**TECANT-1 [µg]**	**% [^{99m}Tc]Tc–TECANT-1 (HPLC) [%]**	**[^{99m}Tc]TcO$_4^-$ [%][a]**	**% ^{99m}Tc-colloid species [%]**	**RCP [%]**
05/20	10	96.3±0.2	<1	4.8±1.6	91.7±1.8
06/20	15	94.5±2.0	<1	4.1±0.4	90.6±1.7
07/20	50	96.4±1.1	<1	5.1±1.1	91.4±0.2

[a]Based on the results of iTLC method described in Table S3.

Table S8. *Direct comparison of labelling batch 01B/21 using two different ^{99}Mo/^{99m}Tc-generators.*

**Generator used**	**Results (mean ± SD)**		
	% [^{99m}Tc]Tc-TECANT-1 (HPLC) [%]	% ^{99m}Tc-colloid species [%]	RCP [%]
Curium generator (n=8)	99.02±0.55	2.74±0.77	96.31±0.89
Polatom generator (n=3)	98.68±0.53	3.23±0.29	95.49±0.26

Figure S1: *Representative radio-TLC chromatogram of [99mTc]Tc-TECANT-1 using 5 M ammonium acetate:methanol (1:1).*

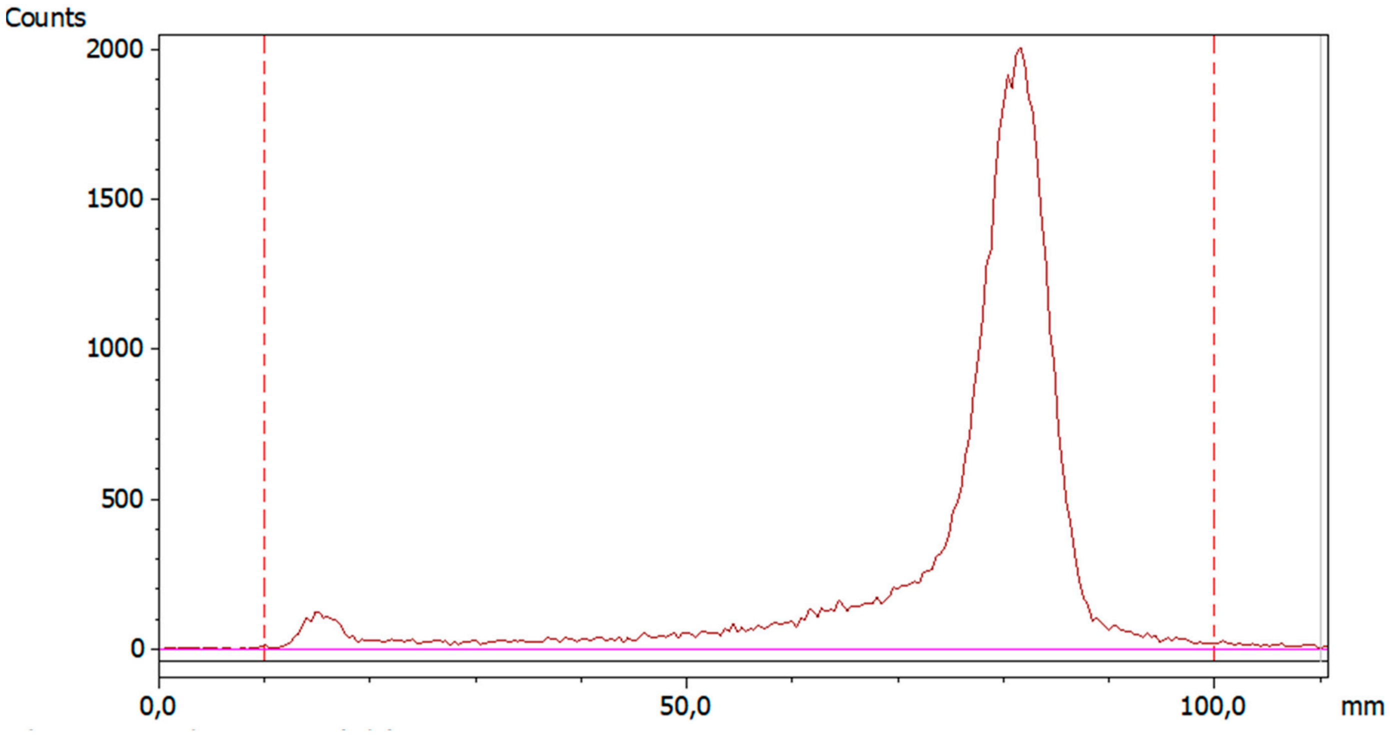

Supplement: Supplementary file 1 [file pharmaceutics-15-00885-s001.zip › pharmaceutics-2202640-supplementary.pdf]
